# Health workforce strategies during COVID-19 response: insights from 15 countries in the WHO Africa Region

**DOI:** 10.1186/s12913-024-10942-z

**Published:** 2024-04-15

**Authors:** Adam Ahmat, Sunny C Okoroafor, James Avoka Asamani, Millogo Jean, Abdou Illou Mourtala, Jennifer Nyoni, Kasonde Mwinga

**Affiliations:** 1 Health Workforce Unit, Universal Health Coverage - Life Course Cluster, World Health Organization Regional Office for Africa, Brazzaville, Congo; 2 Centre for Health Professions Education, North-West University, Potchefstroom, South Africa

**Keywords:** Health workforce, COVID-19, Public health emergencies, Africa, HRH Governance

## Abstract

**Introduction:**

The COVID-19 pandemic unveiled huge challenges in health workforce governance in the context of public health emergencies in Africa. Several countries applied several measures to ensure access to qualified and skilled health workers to respond to the pandemic and provide essential health services. However, there has been limited documentation of these measures. This study was undertaken to examine the health workforce governance strategies applied by 15 countries in the World Health Organization (WHO) Africa Region in responding to the COVID-19 pandemic.

**Methods:**

We extracted data from country case studies developed from national policy documents, reports and grey literature obtained from the Ministries of Health and other service delivery agencies. This study was conducted from October 2020 to January 2021 in 15 countries - Angola, Burkina Faso, Chad, Eswatini, Ghana, Guinea, Guinea Bissau, Ivory Coast, Liberia, Mali, Mauritania, Niger, Nigeria, Senegal and Togo.

**Results:**

All 15 countries had national multi-sectoral bodies to manage the COVID-19 response and a costed national COVID-19 response plan. All the countries also reflected human resources for health (HRH) activities along the different response pillars. These activities included training for health workers, and budget for the recruitment or mobilization of additional health workers to support the response, and for provision of financial and non-financial incentives for health workers. Nine countries recruited additional 35,812 health workers either on a permanent or temporary basis to respond to the COVID-19 with an abridged process of recruitment implemented to ensure needed health workers are in place on time. Six countries redeployed 3671 health workers to respond to the COVID-19. The redeployment of existing health workers was reported to have impacted negatively on essential health service provision.

**Conclusion:**

Strengthening multi-sector engagement in the development of public health emergency plans is critical as this promotes the development of holistic interventions needed to improve health workforce availability, retention, incentivization, and coordination. It also ensures optimized utilization based on competencies, especially for the existing health workers.

## Background

Severe Acute Respiratory Syndrome Coronavirus type 2 (SARS-COV-2), which causes coronavirus disease 2019 (COVID-19) was detected in Wuhan, China in December 2019 and since then, it has been spreading rapidly [1, 2], and mutating to produce different variants of the virus in different parts of the world. In response, the World Health Organization (WHO) declared COVID-19 a public health emergency of international concern on January 30, 2020, and called for collaborative efforts by all countries to contain its spread [3]. As of June 8, 2021, the pandemic has caused more than 3.7 million deaths with at least 172.6 million confirmed cases worldwide [4]. The characteristics of this pandemic are the speed of dissemination, uncertain knowledge, severity, and deaths among caregivers which increase the potential psychological impact on health professionals [5–7]. Globally, 10% of COVID-19 cases were health workers, with the pandemic highlighting the weaknesses in health workforce planning and management, including occupational safety mechanisms in the health systems of countries [8–10].

The cumulative number of COVID-19 cases reported in the WHO Africa region as of June 8, 2021, is 3.5 million with around 3% estimated to be health workers [4]. The cumulative total of COVID-19-related deaths is 88,274 with the regional case fatality rate being 2% [4]. In responding to the pandemic, African governments have made difficult decisions such as the lockdowns, promotion of physical distancing, wearing of face masks or face coverings, early detection, isolation and treatment of those infected with COVID-19 amongst other strategies [11, 12].

Health workers are at the centre of the response globally and they are continually coping with the strain of responding to the pandemic whilst ensuring the continuation of essential health services provision [13, 14]. Studies have suggested that health workers are faced with several risk factors in the course of performing these functions [15, 16]. Not all health workers are adequately trained in the management of COVID-19 patients and there remains inadequate personal protective equipment including masks and other protective equipment [11]. The insufficient numbers of existing health workers are also overwhelmed with heavy workloads due to longer working hours and lack of rest. Furthermore, health workers are also faced with violence and stigma in the workplace, various mental health problems, and emotional distress and burnout [10, 11].

In Africa, the COVID-19 pandemic unveiled huge challenges in health workforce governance in the context of public health emergencies. The pandemic also exacerbated the exposure of health workers to high workloads, hospital-associated infections, violence, stigma, psychological and emotional disturbances, illness, and even death [9]. African governments undertook several measures to address gaps in health workforce surge capacity, health worker protection and ensuring continuity of essential services. However, there has been limited or no documentation of the measures taken and lessons learned.

To provide a more comprehensive view on the impact of the COVID-19 on the health workforce, WHO developed a standardized measurement impact framework [17]. Applying this framework, a series of country case studies were initiated to examine the health workforce governance strategies applied by 15 countries in the WHO Africa Region in responding to the COVID-19 pandemic.

## Methods

### Study design

The overall approach is based on (i) an interim Guidance, developed by WHO in 2020, titled Health workforce policy and management in the context of the COVID-19 pandemic response [18] (ii) the standardized impact measurement framework [17], (iii) and the Health Labor Market Framework. Data was extracted from country case studies developed from national policy documents, reports and grey literature obtained from the Ministries of Health and other service delivery agencies from October 2020 to January 2021. The extraction focused on the content of health workforce plans for COVID-19 response, recruitment of the health workforce for the COVID-19 response, and health workforce mobilization for the COVID-19 response.

### Study setting

This study was conducted from October 2020 to January 2021 in 15 countries - Angola, Burkina Faso, Chad, Eswatini, Ghana, Guinea, Guinea Bissau, Ivory Coast, Liberia, Mali, Mauritania, Niger, Nigeria, Senegal and Togo. These countries had existing country case studies from which information were extracted.

### Data collection

A tool focusing on the COVID-19 response and the health workforce was developed to guide the data extraction from relevant documents. The tool comprised of sections on health workforce planning for COVID-19 response, recruitment of health workforce for the COVID-19 response and health workforce mobilization for the COVID-19 response. These sections were addressed to the following respondents: (1) National bodies for the coordination of the response and case management of COVID-19; (2) Department of Human Resources of the Ministry of Health; (3) Managers of health facilities; and (4) Departments of the Ministry of Health in charge of financial resources, studies and planning.

Secondary data from various sources publicly available and anonymized, including the Human Resources and Public Health Directorates of the Ministries of Health, and the grey literature such as the COVID-19 reportage website, situation reports of the national and sub-national levels, COVID-19 response plans, and reports of national COVID-19 coordination structures were collected.

### Data analysis

Each of the countries analyzed their anonymized data and produced reports which were reviewed by a team of health workforce experts in WHO headquarters and Africa Regional Office. Most of the country reports also underwent stakeholder validation within the country and were cleared by government. The anonymized - quantitative data was extracted from each of the country reports into Microsoft Excel, analyzed and presented in summary in charts.

## Results

The results provide insights on the processes countries applied in planning for the health workforce within the national COVID-19 response plans as well as the strategy employed to ensure health workers are available to provide respond to the pandemic and provide essential health services.

### Health workforce planning within national COVID-19 response plans

To lead the planning and coordination of the COVID-19 pandemic, the 15 countries established national multisectoral bodies. Twelve (12) countries - Angola, Burkina Faso, Chad, Eswatini, Ghana, Guinea, Ivory Coast, Liberia, Mali, Nigeria, Senegal and Togo - also had decentralized planning and coordination structures at the sub-national levels.

All 15 countries that are included in this analysis had costed national COVID-19 response plans. These plans were mostly finalized after the first cases of COVID-19 infection emerged in the countries. All the countries also reflected human resources for health (HRH) activities along the different response pillars (Table 1). Whilst Mauritania, Nigeria and Liberia identified training activities for health workers, Côte d’Ivoire, Liberia and Niger planned and budgeted for the recruitment or mobilization of additional health workers to support the response. All 15 countries provided a range of financial and/or non-financial incentives for health workers, examples are Burkina Faso, Chad and Togo.

**Table 1:** Summary of HRH component of COVID-19 response plans

Country	Summary of how HRH was incorporated into the COVID-19 response plan
Angola	The national response plan for the COVID-19 pandemic incorporated capacity-building activities for health care workers. The process for recruiting 9,290 health professionals in the public sector was exceptionally shortened to ensure that needed health workers were in place within a short space of time.
Burkina Faso	The COVID-19 response and vaccination plans were developed using a multi-sectoral approach. This made it possible to plan and budget for the capacity building and incentives for the health care workers.
Chad	The COVID-19 response plan included major interventions regarding the training, protection and motivation of health and social workers based on estimated needs. The recruitment of 1666 health and social workers during the COVID-19 period was based on the existing recruitment plan in the General State Budget, adopted in 2019. However, the COVID-19 accelerated the resource mobilization and recruitment processes.
Côte D’Ivoire	Côte d‘Ivoire’s National COVID-19 Response Plan addressed the HRH issue from a transversal perspective. For each of its 8 pillars, the specific HRH needs and challenges (recruitments, redeployments, capacity building, etc.) were identified and planned for. To speed up the process of ensuring that health workers are available, the Human Resources Department’s redeployment plan placed 2,740 health workers that were recruited in 2019 at the disposal of the various pillars involved in the fight against the pandemic.
Eswatini	The country incorporated HRH in the national COVID-19 Response Plan and depended on HRH needs forecasting model for recruitments. The plan also focused on shortening bureaucratic processes in recruitment to 30 days and this ensured timely deployment to areas of need.
Ghana	Building on the national COVID-19 response plan, standalone plans for risk communication, case management, surveillance and contact tracing, and HRH were developed. The HRH plan focused on boosting surge capacity through recruitment and equitable distribution, capacity building on COVID-19, and strategies for improving the morale and commitment of staff to respond to the pandemic. Some of the measures included recruitment of additional staff, redeployment of existing staff, insurance cover for health workers and monetary incentives through tax reliefs, and an allowance for all frontline health workers.
Guinea	The National Health Security Agency carried out an estimation of the needs for the health and social workers. The recruitment resulting from this estimate proved to be insufficient to respond to the COVID-19. This informed the recruitment of additional health and social workers on temporary contracts to fill the gap.
Guinea Bissau	The High Commission for COVID-19 repurposed health professionals such as doctors and laboratory staff to support the screening and surveillance services.
Liberia	The incident management system formulated a national COVID-19 preparedness and response plan that considered the health workforce in the public and private sector, and the majority of those mobilized for the COVID-19 response were those that participated in the 2014 Ebola response. The planning and mobilization of the surge team mainly included redeployment of health workers from the county health teams, hospitals, and the national Ministry of Health for the response.
Mali	131 health workers were recruited in 30 days at some hospitals in Bamako, against a usual recruitment process that lasts 90 days. Several regions of Mali developed regional COVID-19 response plans with HRH-related interventions mainstreamed along the pillars. This included a provision for the remuneration of the on-call health workers.
Mauritania	The government recruited additional health personnel for the COVID-19 response. Estimates of needs for this recruitment were made based on the health workforce available in the local health labor market with most of the health assigned to reinforce essential health service delivery.
Niger	The COVID-19 response plan included interventions related to HRH. The development of the plan was based on a multi-sectoral approach, which allowed public authorities from the various sectors to accept and accelerate the recruitment of health workers.
Nigeria	The national COVID-19 response plan mainstreamed HRH interventions in the 8 pillars of the response. The existing health workers particularly doctors, nurses, midwives and medical laboratory scientists were redeployed to provide care in isolation and treatment centers. Health workers were also deployed to support the various response pillars– infection, prevention and control, surveillance, coordination, and risk communication.
Senegal	As part of the response to COVID-19, Senegal strengthened the availability of personnel in health and social action structures. Temporary recruitments of 770 health workers was done, with the duration of the recruitment process being 14 days against the usual 60 days. In addition, redeployments were carried out for 45 doctors, and 364 students and retired health workers.
Togo	The process of developing the COVID-19 response plan was inclusive. HRH was an integral part of the different strategies developed and implemented and this included the recruitment of temporary staff (712 medical staff and 996 paramedical staff), the development of national and sub-national training plans, incentivizing for staff involved in the management of COVID-19 cases etc. Actions relating to HRH represented 23% of the Plan’s budget.

*Other health workers includes Pharmacists, pharmacy technicians and pharmacy assistants, Medical and pathological laboratory technologists and technicians, Technicians of Health information and health data entry officers, Community health workers, Health workers and hygiene and sanitation technicians, Medical imaging or therapeutic equipment technicians etc.

*Other health workers includes Pharmacists, pharmacy technicians and pharmacy assistants, Medical and pathological laboratory technologists and technicians, Technicians of Health information and health data entry officers, Community health workers, Health workers and hygiene and sanitation technicians, Medical imaging or therapeutic equipment technicians etc.

Two countries (Eswatini and Ghana) developed stand-alone or HRH plans for COVID-19 which was an extension of the national COVID-19 response plan while others had applied the existing HRH plans in supporting the COVID-19 response in partnership with the human resource departments. At the time of developing initial COVID-19 response plans none of the countries estimated the surge capacity required for the response.

### Recruitments

**Fig. 1:**
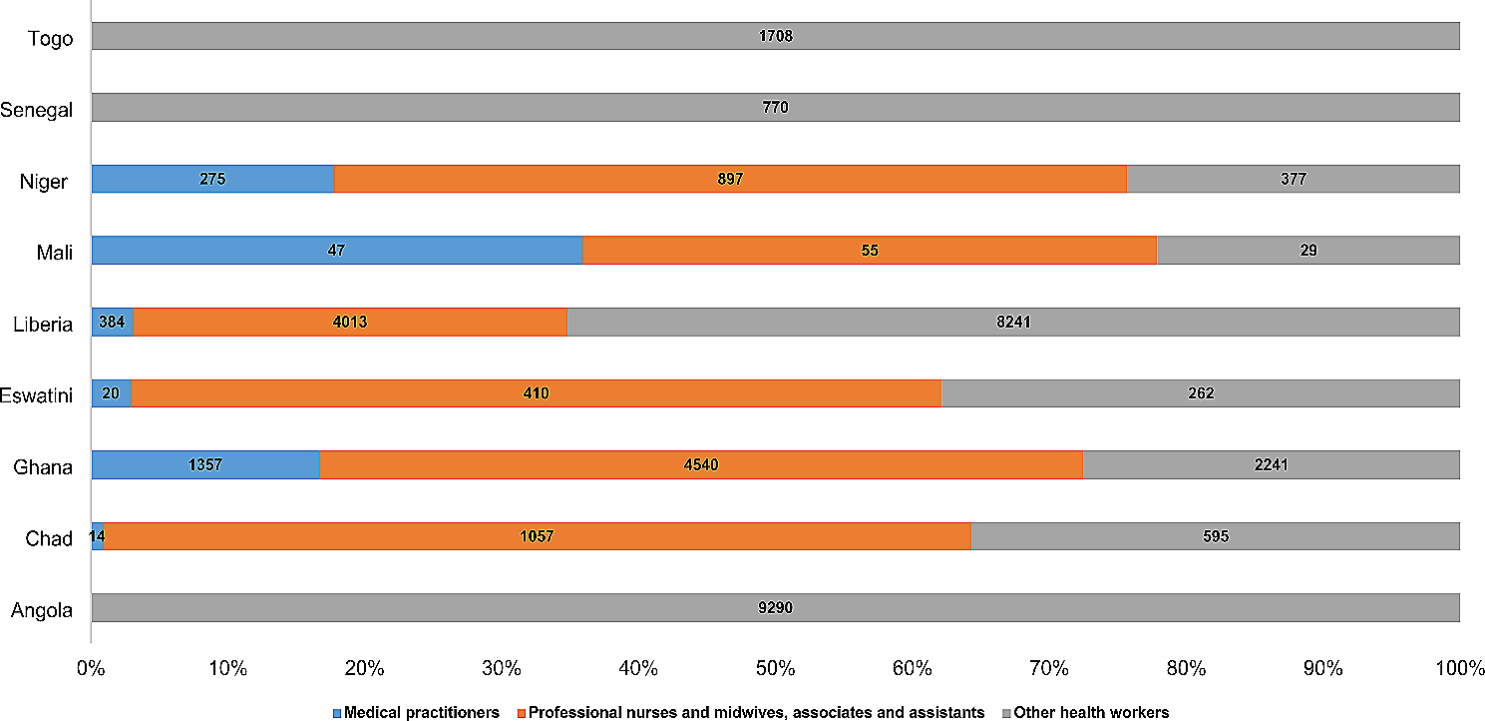
Distribution of health personnel recruited in 9 countries for COVID-19 response

Nine countries– Angola, Chad, Ghana, Eswatini, Liberia, Mali, Niger, Senegal and Togo- recruited additional health workers either on a permanent or temporary basis to respond to the COVID-19. As shown in Fig. 1, a total of 35,812 health workers were recruited in these countries. The recruitment process did not follow the usual norm of advertisement, shortlisting and evaluation of candidates. An abridged process that reduced the timeframe for recruitment was taken to ensure that needed health workers were in place for deployment. Ghana (65%, *n* = 1357) recruited the most medical practitioners followed by Liberia (18%, *n* = 384), Niger (13%, *n* = 275), Mali (2%, *n* = 47), Eswatini (1%, *n* = 20) and Chad (1%, *N* = 14). For nurses and midwives, 6 countries– Ghana (41%, *n* = 4540), Liberia (37%, *n* = 4013), Chad (10%, *n* = 1057), Niger (8%, *n* = 897), Eswatini (4%, *n* = 410) and Mali (1%, *n* = 55) recruited a total of 10,972 practitioners. Angola (41%, *n* = 9290), Liberia (36%, *n* = 8241), Ghana (10%, *n* = 2241), Togo (8%, *n* = 1708), Senegal (93%, *n* = 770), Chad 93%,*n* = 595) and Eswatini 91%, *n* = 262) recruited 22,743 of other categories of health workers including pharmacists, pharmacy technicians and pharmacy assistants, Medical and pathological laboratory technologists and technicians, Technicians of Health information and health data entry officers, Community health workers, Health workers and hygiene and sanitation technicians, Medical imaging or therapeutic equipment technicians.

### Redeployment of health workers

The initial redeployment of existing staff in all 15 countries was not done based on methodologies or tools for estimating health workforce needs, rather based on perceived need. In Angola, Burkina Faso, Ghana, Ivory Coast, Senegal and Togo where existing 3671 health workers were redeployed to respond to the COVID-19, these redeployments, were carried out to strengthen the provision of COVID-19 case management services. A total of 3,671 health workers were redeployed in six countries– Angola (47%, *n* = 1718), Burkina Faso (1%, *n* = 47), Ghana (4%, *n* = 148), Ivory Coast (32%, *n* = 1165), Senegal (11%, *n* = 419) and Togo (5%, *n* = 174).

**Fig. 2:**
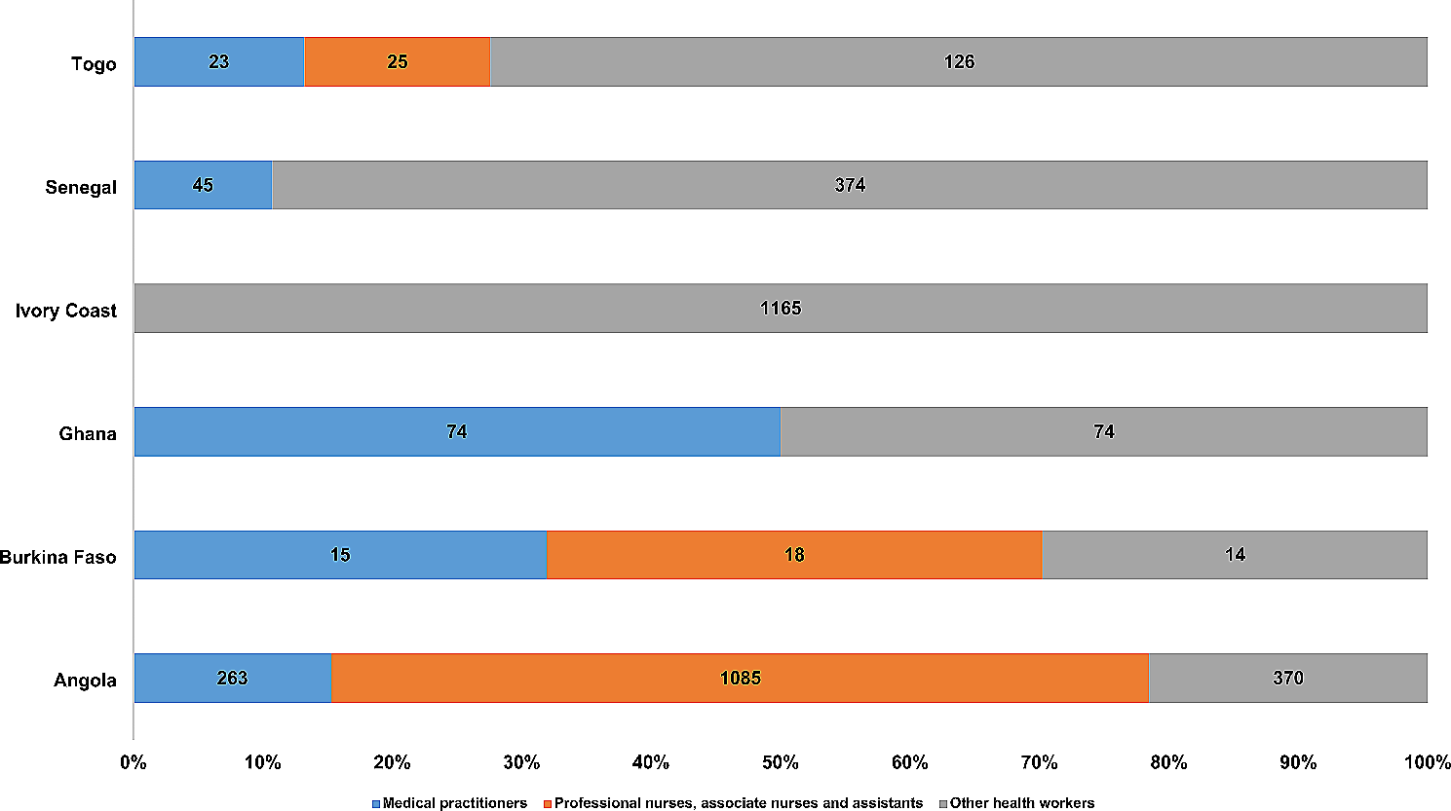
Distribution of redeployments for better management of COVID-19 patients

As shown in Fig. 2, Angola redeployed 263 (15%) medical practitioners, 1085 (63%) nurses and 370 (22%) of other categories of health workers, and Burkina Faso redeployed 15 (32%) medical practitioners, 18 (38%) nurses and 14 (30%) of other categories of health workers to support the COVID-19 case management in the countries. In Togo, 13% (*n* = 23), 14% (*n* = 25) and 72% (126) of medical practitioners, nurses and other categories of health workers were redeployed to support the COVID-19 service delivery across the country.

National reports indicated that the redeployment of existing health workers was indicated to have negatively affected the continuity of essential health service provision. Suggested reasons included that health facilities were dispossessed, temporarily or permanently, of some of their key staff resulting in high workloads and closure or absence of some.

## Discussion

This paper examined the health workforce governance strategies applied by 15 countries in the WHO Africa Region in responding to the COVID-19 pandemic. This study indicates that HRH development activities were included in the COVID-19 response plans along the response pillars, especially case management, infection prevention and control, and risk communication. The planned activities included training of health workers, protection and recruitment or mobilization of additional health workers to support the response. Few countries planned for bonuses and other financial or non-financial benefits for health workers responding to COVID-19.

Health workforce planning is essential for health emergency pillars such as preparedness, response and recovery to ensure that sustainable essential health services are provided during public health emergencies including the COVID-19 pandemic. Moreover, health workforce planning ensures that a strategic approach is taken to ensure that the health workforce contributes maximally to ensuring health care services are provided and the health of the population is improved [14, 19]. It also ensures that plans are put in place to ensure that health worker safety is ensured as this is important in facilitating effective service delivery. Additionally, it reduces the evident understaffing which resulted in most health workers being exhausted and facing mental health issues whilst responding to the pandemic or providing essential health services. Also needed is the training of the health workforce to ensure that the relevant skills needed for specific emergencies are put in place for quality response [20].

In planning for the health workforce in the context of public health emergencies like the COVID-19 pandemic, a whole-of-society approach is key considering that other ministries and stakeholders have roles in the management and funding of the health workforce [14]. A multi-sectoral approach to health workforce planning ensures that varied institutions pull resources at their disposal towards common objectives aimed at mitigating the impact of public health emergencies [19]. Consequently, this mitigates policy formulation and implementation barriers and assure scaled-up response with distinct sectors striving hard towards the accomplishment of common health goal that would otherwise be difficult for a single sector to achieve alone.

This study indicated that countries shortened the recruitment processes for health workers by applying a coordinated and consensual approach with other sectors to ensure needed staff were in the right place on time– this is one of the key lessons learned in these countries. Mobilizing and deploying health workers with the active involvement of the Directorate of HRH further improved the availability and distribution of the health workforce at the COVID-19 clinics. Effective deployment played a key role in ensuring improved health outcomes.

Planning and implementing financial and non-financial incentives for health workers is beneficial in motivating them to perform optimally whilst responding to public health emergencies [21]. Enhanced working conditions and environment also motivate them and improve retention [22]. To protect the families of health workers, risk assessments should be conducted with support provided to those that may need to either self-isolate or quarantine in the course of providing services to protect their families.

Estimating health workforce needs for public health emergencies is important as this ensures that numbers, skills and competencies are appropriate to respond effectively. However, this should be determined based on context as the health workforce needs would vary from one country to another. Furthermore, certain skills such as leadership skills, problem-solving skills, strategic skills, and communication skills are highly essential for the health workers whilst serving the needs of the people in the context of public health emergencies. Consequently, the skills of the health workforce for public health emergencies are critical for improved response [23].

Applying evidence-based approaches for the deployment or redeployment of health workers in the context of public health emergencies is key. Using evidence-based response parameters of interest in deploying or redeploying health workers during public health emergencies promotes mitigation impact uniformly and improves the efficiency in health service delivery [24].

Also important is learning from the public health emergency to improve resilience of the entire health system for future ones [25, 26]. Several strategies, including those above, have been highlighted in the literature. Implementing these strategies should be guided by core principles which are government ownership and leadership, whole-of-government and whole-of-society approaches, ensuring equity by leaving no one behind, sustaining essential health service delivery whilst managing health emergencies, engaging and empowering communities, and learning from previous emergencies to improve policies and interventions [25]. The key strategies include fostering whole-of-society engagement to collectively address health challenges and reorganizing health systems to prioritize Primary Health Care (PHC). Investing in the Essential Public Health Functions (EPHF) serves as a foundational strategy, ensuring robust public health infrastructure with capabilities in disease surveillance, health promotion, and emergency preparedness. Addressing existing inequities in access to health services ensures inclusivity during emergencies. Sustainable financing is recognized as essential for stability, while creating an enabling environment for data generation, sharing, research, innovation, and learning underscores the importance of informed decision-making and adaptability in the face of evolving health challenges [25, 26]. Implementing these comprehensive strategies would fortify national health systems, making them more resilient, equitable, and responsive to population needs.

### Limitations

We planned to present the health workforce strategies applied in the 47 countries in the WHO Africa Region as part of the pandemic COVID-19 responses. However, only 15 countries agreed to participate in this process initially with issues on the need for data confidentiality being highlighted in most countries. Thus, the findings are not representative of the region. We are also aware that the response mechanisms continued to change after data was collected as countries continued to change strategies to respond to the evolving pandemic. Also important to note is that data management for the health workforce responding to the COVID-19 pandemic was not readily available, completed and centralized. Thus, the numbers of health workforce recruited in some countries were often not properly enumerated and disaggregated.

## Conclusion

As health workers are core to public health emergency response, health workforce planning should be an integral part of public health emergency prevention, preparedness, response and recovery plans. A whole-of-government approach is important to ensure that all sectors contribute to achieving the set goals. Also, through a coordinated approach to mobilizing health workers in partnership with the sectors responsible for recruitment, deployment, education and regulation of health workers, the suitable distribution, competencies, and skill mix needed for the response would be available.

## Data Availability

The datasets used and/or analysed during the current study available from the corresponding author on reasonable request.
